# 55 Assessing Pediatric Burn Wound Infection Using a Point-of-Care Autofluorescence Imaging Device

**DOI:** 10.1093/jbcr/irae036.047

**Published:** 2024-04-17

**Authors:** Evan Turner, Jennifer Zuccaro, Hawwa Chakera, Charis Kelly, Joel Fish

**Affiliations:** University of Toronto, Toronto, Ontario; Hospital for Sick Children, Toronto, ON; University of Toronto, Toronto, Ontario; Hospital for Sick Children, Toronto, ON; University of Toronto, Toronto, Ontario; Hospital for Sick Children, Toronto, ON; University of Toronto, Toronto, Ontario; Hospital for Sick Children, Toronto, ON; University of Toronto, Toronto, Ontario; Hospital for Sick Children, Toronto, ON

## Abstract

**Introduction:**

Wound infection is the most common complication among pediatric burn patients and when not treated promptly, delayed healing, failure of skin grafts, or death can result. Standard burn wound assessment includes inspection for visual signs and symptoms of infection and microbial sampling. To aid in the assessment of burn wound infection, a point-of-care autofluorescence imaging device was introduced at the study institution in 2020. This imaging device uses violet light to illuminate the wound bed causing clinically relevant quantities of bacteria to fluoresce in real-time. The objectives of this study were to evaluate the role of the autofluorescence imaging device in the management of pediatric burn wounds and determine if the imaging findings corresponded to visual signs and symptoms of infection and/or microbial sampling.

**Methods:**

A retrospective review of patients aged 0-18 years who had their burn wounds assessed with the autofluorescence imaging device between 2020-11-01 and 2023-06-08 was conducted. All imaged wounds were inspected for visual signs and symptoms of infection and had swabs collected for the purpose of microbial sampling. Sensitivity and specificity analyses were carried out on a subset of wounds that were imaged after initial wound cleaning was performed.

**Results:**

Data were extracted from the medical records of 178 eligible burn patients with 218 wounds imaged. The mean age of patients was 3.2 years (SD 3.7), and most burns were partial thickness (78%) and due to scalds (81%). Fluorescence was detected by imaging in 16% of wounds, while 11% of wounds had visual signs and symptoms of infection and 16% had positive wound swab findings. Autofluorescence imaging corresponded with visual signs and symptoms of infection in 81% of wounds and microbial findings in 82% of wounds. Sixty-three patients with 77 wounds were included in sensitivity and specificity analyses. Relative to visual signs and symptoms of infection alone, combining autofluorescence imaging with visual signs and symptoms of infection resulted in a 39% increase in sensitivity and 19% decrease in specificity.

**Conclusions:**

Autofluorescence imaging correlates well with visual signs and symptoms of infection and microbial sampling in pediatric burn wounds and complements inspection for visual signs and symptoms of infection by improving detection.

**Applicability of Research to Practice:**

Incorporation of this autofluorescence imaging device in standard burn wound assessments can augment identification of wound infections, which can enhance diagnostic confidence at the point-of-care and ultimately improve wound healing outcomes.

